# Lipidomic and transcriptomic remodeling underlies early retinal pathogenesis in dry age-related macular degeneration

**DOI:** 10.1038/s41419-026-09147-5

**Published:** 2026-08-07

**Authors:** Neeta Roy, Jen-Zen Chuang, Ting-Huan Chen, Ye Sun, Danielle E. Allen, Marie-Audrey I. Kautzmann, Surjyadipta Bhattacharjee, Maxwell Ping Lee, Laura Beth J. McIntire, Nicolas G. Bazan, Ching-Hwa Sung

**Affiliations:** 1 Department of Ophthalmology, Margaret M. Dyson Vision Research Institute, Weill Cornell Medicine, New York, NY USA; 2 Neuroscience Center of Excellence, School of Medicine, Louisiana State University Health Sciences Center, New Orleans, LA USA; 3 The Laboratory of Cancer Biology and Genetics, Center for Cancer Research, National Cancer Institute, National Institutes of Health, Bethesda, MD USA; 4 Department of Radiology, Weill Cornell Medicine, Brain Health Imaging Institute, New York, NY USA; 5 Department of Cell and Developmental Biology, Weill Cornell Medicine, New York, NY USA

**Keywords:** Lipids, Neurodegeneration

## Abstract

Dry age-related macular degeneration (AMD) is initiated by retinal pigment epithelium (RPE) dysfunction, yet early metabolic mechanisms linking RPE injury to photoreceptor dysfunction remain unclear. Using a physiologically relevant mouse model, we performed cell-type–resolved lipidomic and transcriptomic profiling with ultrastructural analyses. RPE-specific loss of Chloride Intracellular Channel 4 (CLIC4) induced coordinated lipid remodeling and transcriptional rewiring compromising RPE–photoreceptor lipid homeostasis. Both RPE and neural retina exhibited shifts toward very-long-chain unsaturated phospholipids and ceramides, while mutant RPE also accumulated saturated very-long-chain ceramides and cholesterol, particularly in females, favoring drusen deposition. Strikingly, RPE and rods mounted divergent responses: RPE activated compensatory mechanisms, whereas rods exhibited transcriptomic and ultrastructural evidence of bioenergetic suppression, mitochondrial structural abnormalities, and mitochondria-derived oxidative damage, establishing a feed-forward stress cycle extending beyond canonical rod-RPE carbon-energy coupling. These findings underscore rod mitochondrial structural integrity as a potential early biomarker, positioning disrupted lipid recycling as a targetable axis in dry AMD pathogenesis.

## Introduction

Age-related macular degeneration (AMD) is the leading cause of irreversible vision loss in the elderly, and its predominant form, dry AMD, lacks effective therapy. Although the retinal pigment epithelium (RPE) has long been considered the primary site of pathology, extensive RPE loss occurs only in advanced geographic atrophy. In contrast, the earliest functional hallmarks of disease are delayed rod-mediated dark adaptation and selective rod vulnerability [1–3]. This temporal dissociation suggests that early metabolic dysfunction precedes overt cell loss and may drive disease initiation.

Converging lines of genetic and epidemiological evidence point to dysregulated lipid metabolism as a key player in AMD pathogenesis. Variants in genes encoding lipid transport and metabolism proteins, including *APOE*, *ABCA1*, *LIPC*, and *CETP*, have been linked to AMD risk. The pathological hallmark of dry AMD, drusen, are lipid-rich deposits within Bruch’s membrane that are heavily enriched in cholesteryl esters and ceramides [4–6]. Excess lipid droplets (LDs) and intracellular cholesterol accumulation have been documented in donor eyes [7–11], and human studies have reported altered circulating ratios of docosahexaenoic acid (DHA; 22:6n-3) to arachidonic acid (AA; 20:4n-6) in AMD patients [12], alongside reduced retinal very-long-chain polyunsaturated fatty acid (VLC-PUFA) content in donor eyes [13].

Together, these observations have led to the proposal that dysregulated lipid metabolism contributes to AMD by promoting oxidative stress, triggering inflammatory cascades, and driving drusen deposition. Yet the mechanistic relationship between these processes remains to be established. Furthermore, the temporal relationship between lipid imbalance and rod photoreceptor dysfunction, as well as the cellular origins and molecular mechanisms governing pathological lipid accumulation, represents critical gaps that must be addressed to fully understand AMD pathogenesis.

The retina is one of the most lipid-rich tissues in the body, owing to the highly specialized membranes of photoreceptor outer segments (OS). These membranes are enriched in DHA and VLC-PUFAs, which confer structural flexibility and support phototransduction [14, 15], but their abundance of double bonds renders them highly susceptible to oxidative damage. Lipid homeostasis in the outer retina relies on a tightly coordinated metabolic circuit: dietary DHA is taken up by the RPE, incorporated into phospholipids in the RPE, and then transferred to photoreceptors for OS biogenesis, and recycled through daily RPE phagocytosis [16–18]. VLC-PUFAs are elongated in photoreceptors and subsequently metabolized in the RPE via peroxisomal β-oxidation, while the RPE exports cholesterol for systemic clearance. This bidirectional lipid flux maintains RPE–photoreceptor metabolic coupling, and disruption of this circuit can compromise photoreceptor integrity and promote sub-RPE lipid deposition.

**Fig. 1:**
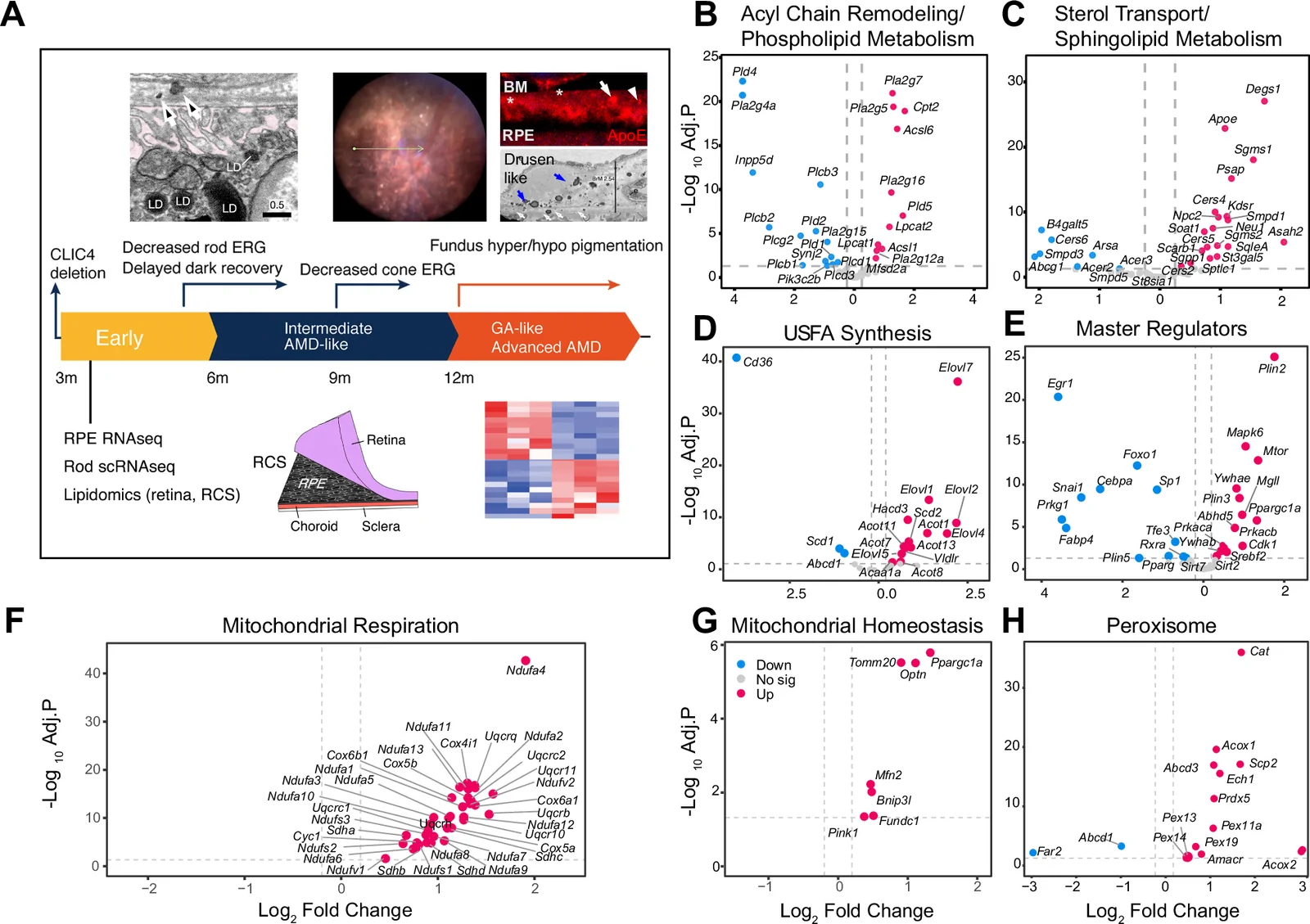
Temporal progression of AMD-like pathology and transcriptional changes in RPE^∆Clic4^ mice. **A** Schematic illustrating the temporal sequence of AMD-like clinical and histopathological features observed in RPE^∆Clic4^ mice [19], with corresponding timelines for tissue collection for bulk RNA-seq of purified RPE and scRNA-seq of retina. **B**–**H** Volcano plots showing significantly altered genes (FDR ≤ 0.05) in indicated pathways in purified RPE from KO versus Ctrl mice (*N* = 3 each genotype) Each panel highlights DEGs associated with specific functional pathways affected by *Clic4* loss.

Characterizing the temporal relationship between lipid remodeling, visual functional decline, and drusen formation, and their causal role in AMD initiation, relies on a validated animal model. An RPE-specific Chloride Intracellular Channel 4 (CLIC4) knockout mouse line, RPE^∆*Clic4*^ (hereafter KO), was recently established and recapitulates a full spectrum of functional, clinical, and histological phenotypes resembling dry AMD in a temporal sequence paralleling the progression from intermediate to advanced disease [19] (Fig. 1A). These include early fundus abnormalities, progressive rod-then-cone dysfunction, accumulation of intracellular lipid droplets, sub-RPE/Bruch’s membrane deposition of APOE⁺/complement C3⁺/Oil Red O⁺ drusen-like material, and eventually extensive RPE cell death resembling late-stage geographic atrophy. Notably, CLIC4-deficient RPE cells exhibit elevated 4-hydroxynonenal (4-HNE) adducts, indicating early lipid oxidative stress [19].

Bulk RNA-seq and pathway-enrichment analyses, including Ingenuity Pathway Analysis (IPA), performed on validated and purified RPE cells from pre-symptomatic (3-month-old) KO versus *Clic4* floxed control (Ctrl) mice revealed dysregulation of multiple pathways implicated in human AMD pathogenesis, including lipid metabolism, oxidative phosphorylation, and inflammatory signaling [19]. These findings further highlight the utility of this model for investigating how early lipid alterations contribute to retinal vulnerability and AMD progression.

Despite its name, CLIC4 is not a conventional transmembrane ion channel; rather, it is a cytosolic or membrane-associated protein with a glutathione S-transferase–like fold [20] and antioxidant properties [21]. CLIC4 localizes to the plasma membrane as well as multiple intracellular compartments, including endoplasmic reticulum, endosomes, mitochondria, and the nucleus, where it performs context-dependent functions [21, 22]. In RPE cells specifically, CLIC4 has been implicated in endosomal vesicular trafficking, extracellular matrix remodeling [23], F-actin cytoskeletal organization [24], and transcriptional regulation [19]. The molecular mechanisms by which CLIC4 deficiency contributes to cell-autonomous and non-cell-autonomous AMD pathogenesis, however, remain incompletely understood.

In this study, we integrated cell-type–resolved transcriptomic profiling and targeted lipidomic analysis of RPE^∆*Clic4*^ mice at 3 months of age, before the onset of electrophysiological decline at 6 months (Fig. 1A), to define early metabolic rewiring events linking RPE dysfunction to photoreceptor injury. Collectively, these findings identify RPE lipid dysregulation, photoreceptor mitochondrial dysfunction, and oxidative stress as mechanistically integrated components of early AMD pathogenesis.

## Results

### Transcriptomics uncovers lipid remodeling and metabolic reprogramming mechanisms in CLIC4-deficient RPE

Target gene analysis of differentially expressed genes (DEGs) from bulk RNA-seq data of purified RPE cells isolated from KO and Ctrl mice revealed expression changes in key components of lipid remodeling, biosynthesis, trafficking, and metabolic regulation pathways (Table S1). The direction and magnitude of these changes are illustrated in heatmaps (Fig. S1A) and volcano plots (Fig. 1B–E).

Together, these analyses point to a coordinated, system-level transcriptional reprogramming characterized by concurrent alterations in lipid turnover and lipid synthesis. With respect to lipid turnover, we observed downregulation of *Cd36*, which is essential for photoreceptor OS phagocytosis and FA uptake [25], and *Pla2g15* (Fig. 1B), as well as *Pla2g15* (Fig. 1B), which encodes the lysosomal PLA2 required for phagolysosomal clearance. Reduced *Abcd1* expression (Fig. 1D), a peroxisomal VLCFA transporter, may further impair VLCFA degradation. Suppression of components of the FABP4–C/EBPα–SP1 regulatory axis (Fig. 1B, D, E) is also consistent with reduced transcriptional programs associated with FA uptake and lipid trafficking. Reciprocal changes in perilipin expression further support altered lipid handling: increased *Plin2* and *Plin3* levels (Fig. 1E) are consistent with enhanced LD accumulation, whereas decreased *Plin5* expression may impair lipolysis by disrupting the coupling of LDs to mitochondrial FA β-oxidation [26, 27].

Transcriptional remodeling characterized by impaired cholesterol efflux and increased cholesterol synthesis, favoring net cholesterol accumulation, was also observed. *Abcg1* (Fig. 1C), which encodes a cholesterol efflux transporter that normally co-localizes with ABCA1 at the basal RPE plasma membrane, and *Npc2* and *Apoe*, which involved in lysosomal cholesterol efflux and alternative export pathways, respectively, were all downregulated. Consistent with IPA-identified activation of cholesterol synthesis pathways (Fig. S1C, Table S2), several key biosynthetic genes were upregulated, including *Sqle*, which encodes the rate-limiting enzyme in cholesterol biosynthesis, *Srebf2*, a master regulator of cholesterol biosynthesis and uptake, and *Soat1* (also known as *Acat1*), which encodes a cholesterol esterification enzyme.

Multiple Elovl family members (*Elovl1*, *Elovl4*, *Elovl5*, *Elovl7*) were upregulated (Fig. 1D), reflecting enhanced FA elongation activity. In parallel, ceramide synthase expression was altered, including upregulation of *Cers2*, *Cers4*, and *Cers5*, and downregulation of *Cers6* (Fig. 1C), collectively favoring the synthesis of VLC ceramides. These transcriptional changes are consistent with the lipid species alterations observed in the lipidomics analyses described below.

IPA predicts inhibition of mTOR and p70S6K signaling pathways (Supplementary Fig. S1C, Table S2). Inhibition of mTORC1, a key suppressor of autophagy and mitophagy, is consistent with the upregulation of multiple mitophagy-related genes, including *Pink1*, *Bnip3l*, *Fundc1*, and *Mfn2* (Fig. 1G). Although *Mtor* transcripts were increased, this likely reflects a compensatory transcriptional response, as mTOR signaling is primarily regulated at the post-transcriptional and post-translational levels.

Upregulation of *Ppargc1a*, encoding PGC-1α (Fig. 1G), along with several mitochondrial electron transport chain (ETC) components (Fig. 1F), suggests enhanced mitochondrial biogenesis and remodeling. PGC-1α may further co-activate PPARα-dependent programs to promote FA oxidation (Table S2), consistent with the upregulation of multiple FA oxidation genes, including *Acaa1*, *Acadl*, and *Acox1*. Whether this transcriptional activation translates to effective FA utilization, however, may be limited by impaired LD-mitochondria coupling, as discussed above.

### Enhanced VLC-PUFA incorporation and DHA redistribution in KO retina and RCS

**Fig. 2:**
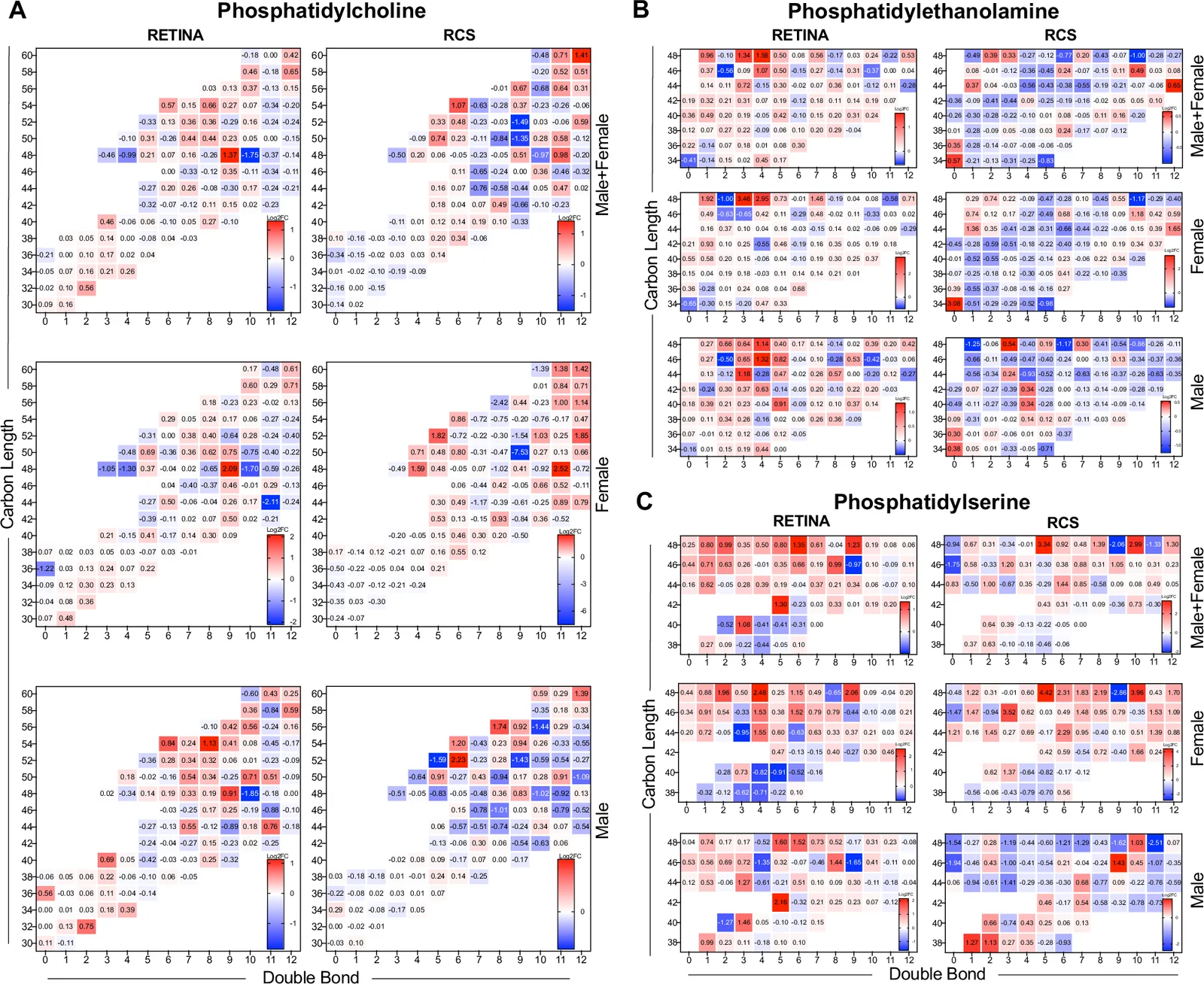
Altered phospholipid profiles in retina and RCS of RPE^∆Clic4^ mice. Heatmaps display fold changes in PC (**A**), PE (**B**), and PS (**C**) species in retina and RCS tissues from KO mice compared to Ctrl. The x-axis represents the number of double bonds, and the y-axis shows the carbon chain length of each lipid species. Fold-change (log_2_FC) reflected within each heatmap cell are obtained from mean of % expression of lipid species within each lipid group in KO as compared to Ctrl. Data are derived from *N* = 6 Ctrl and *N* = 6 KO samples in the mixed-sex cohort, with sex-stratified analyses including 3 female and 3 male samples per group.

Targeted lipid species were profiled by LC–MS/MS in the neural retina and the RPE–choroid–sclera (RCS) complex, mechanically isolated from 3-month-old mice. Lipid classes analyzed included phosphatidylcholine (PC) (Figs. 2A, S2A, B), phosphatidylethanolamine (PE) (Figs. 2B, S3A, B), phosphatidylserine (PS) (Figs. 2C, S4A, B), VLCFAs (Fig. S5), sphingomyelins (Fig. S6), ceramides (Figs. 4A–E, S7A, B), and cholesterol and oxysterols (Figs. 4G, S8A, B). Six mice (three males and three females) per genotype, KO and Ctrl, were analyzed. Changes in species are shown. Lipid species are presented as percentages of total lipids within each class. Corresponding heatmaps depict relative fold changes for all assayed lipid species, with selected species highlighted in bubble plots. All quantitative values are also provided in bar graphs in the Supplementary Figures.

**Fig. 3:**
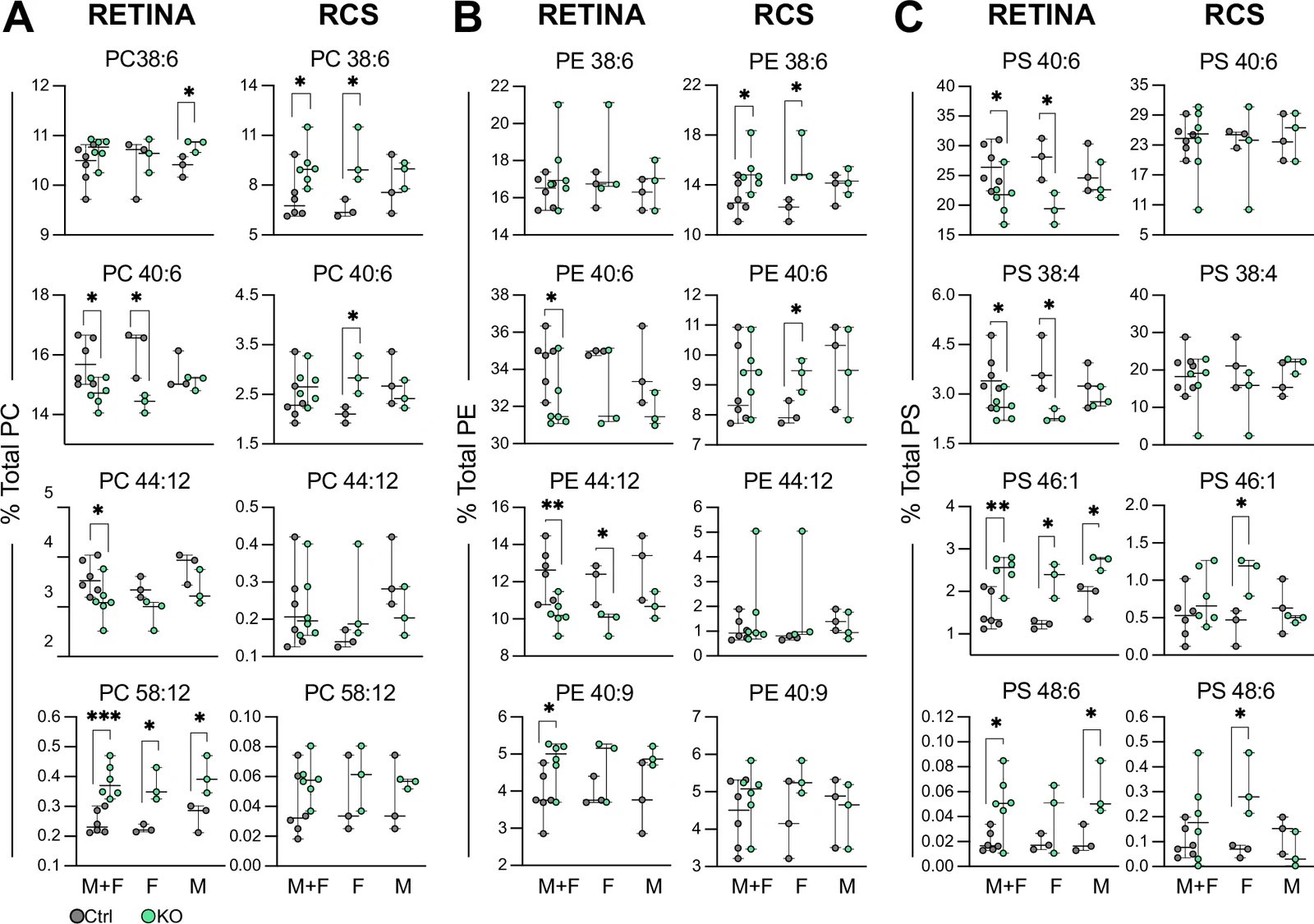
Altered DHA-, PUFA- and AA-containing phospholipids in retina and RCS of RPE^∆*Clic4*^ mice. Representative plots showing alterations in PC (**A**), PE (**B**), PS (**C**) in retina and RCS of KO mice relative to Ctrl, analyzed in both mixed-sex and sex-stratified groups. Data are from *N* = 6 Ctrl and *N* = 6 KO samples in the mixed-sex cohort, and *N* = 3 Ctrl and *N* = 3 KO samples per sex in sex-stratified analyses. Statistical significance was assessed by two-tailed Student’s t-test; *p ≤ 0.05, **p < 0.01. Source data of (**A**–**C**) are provided as a Source Data file.

Initial mixed-sex analysis revealed increased VLC-PUFA–containing PC species in KO retinas (Fig. 2A), with PC58:12 reaching significance (Fig. 3A), consistent with enhanced VLC acyl incorporation. Sex-stratified analysis confirmed a broad elevation of VLC-PUFA PCs in both females and males (Figs. 2A, 3A). Canonical DHA-containing PCs, however, exhibited a sex-specific redistribution. In females, retinal levels of PC40:6 and PC44:12 were reduced, accompanied by reciprocal increases in the RCS (Fig. 3A). In males, this pattern was absent; instead, a selective increase in retinal DHA-PC species PC38:6 was observed, without a corresponding RCS shift.

Mirroring the PC profile, mixed-sex analysis revealed a trend toward increased VLC-PUFA–containing PE species in KO retinas (Fig. 2B), with several reaching statistical significance, including PE40:9 (Fig. 3B), PE40:7, PE44:3, PE48:3, and PE48:4 (Fig. S3A). Sex-stratified analysis recapitulated PC patterns: female KO retinas exhibited a clear reciprocal redistribution, with DHA-containing PEs (PE44:12, PE40:6) reduced in retinas and increased in the RCS (PE38:6, PE40:6) (Fig. 3B). In males, retinal DHA-PE species (PE44:12, PE40:6) showed a trend of reduction, but the RCS did not show corresponding elevations. Remodeling of PE lipids in OS membranes may alter the pool of PE available for bis-retinoid formation and lipid peroxidation, potentially influencing A2E/lipofuscin accumulation and RPE stress, processes implicated in retinal pathology [28, 29].

Although less abundant than other phospholipids, PS plays an essential role in inner leaflet membrane curvature, signaling, mitochondrial PE synthesis, and respiratory chain function [30]. In mixed-sex analyses, KO retinas exhibited a shift toward VLC unsaturated PS species (Fig. 2C), including PS46:1, PS48:6 (Fig. 3C), PS42:5, and PS44:1 (Fig. S4A). While female KO retinas also showed a reduction in DHA-containing PS40:6, a corresponding increase in the RCS was absent (Fig. 3C).

Mixed-sex analyses showed a shift toward VLC-unsaturated PS species in KO RCS retinas (Fig. 2C), largely driven by females with significant increases in PS46:1, PS48:6 (Fig. 3C), and PS42:8 (Fig. S4B). In contrast, males generally exhibited a decreasing trend, with few exceptions (Figs. 2C, S4B). Notably, AA–containing PS38:4 was decreased in retinas of both sexes (Fig. 3C), while AA-containing PC and PE species remained unchanged in both retina and RCS.

In KO mice, VLC-PUFAs (FA32:6n-3, FA34:6n-3) and their oxygenated intermediates (27-monohydroxy-32:6, 29-monohydroxy-34:6), which serve as precursors for retinal-specific neuroprotective elovanoids (Fig. S5A), exhibited modest, non-significant downward trends in both retina and RCS (Fig. S5B-E).

### VLC-Ceramide and Free Cholesterol Enrichment in KO RCS

Sphingolipids, including sphingomyelin and ceramide, regulate membrane microdomain organization and cellular signaling. Sphingomyelin species were not significantly altered in either the retina or RCS across sexes, partly due to inconsistent directional changes observed between males and females (Fig. S6A–C).

**Fig. 4:**
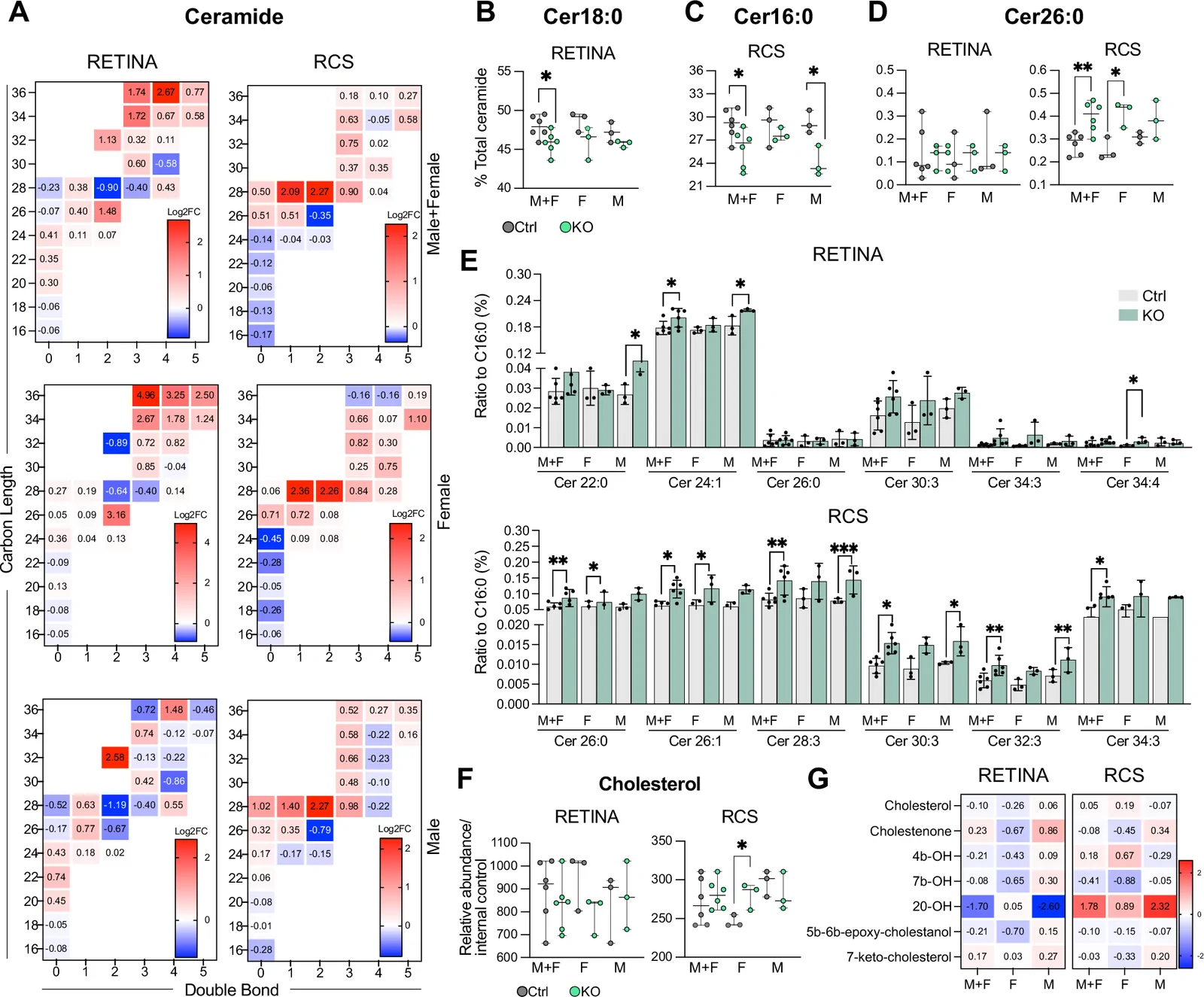
Remodeling of sphingomyelin, ceramide, and cholesterol in retina and RCS of RPE^∆*Clic4*^ mice. **A** Heatmaps showing fold changes in ceramide species in KO vs. Ctrl samples. **B**–**D** Representative plots illustrating changes in indicated ceramide species in mixed-sex and sex-stratified analyses. **E** Histogram showing the abundance of selected ceramide species related to Cer16:0. **F** Graph depicting changes in free cholesterol levels. **G** Heatmaps showing fold changes in cholesterol and oxysterol species in KO vs. Ctrl samples. Data are from *N* = 6 Ctrl and *N* = 6 KO samples in the mixed-sex cohort, and *N* = 3 Ctrl and *N* = 3 KO samples per sex in sex-stratified analyses. Statistical significance was determined using a two-tailed Student’s t-test; *p ≤ 0.05, **p < 0.01, ***p < 0.001. Source data of (**B**–**F**) are provided as a Source Data file.

In contrast, the ceramide pool in KO RCS showed a marked shift. In retina and RCS, both mixed-sex and sex-stratified analyses showed a trend toward reduced Cer16:0 and Cer18:0 (Figs. 4A, S7A, B), the most abundant ceramides in retinal tissues, with significant decreases of Cer18:0 in the retina and Cer16:0 in the RCS (Fig. 4B, C). This was unexpected, as elevated Cer16:0 is typically associated with apoptosis [31]; its relative reduction suggests minimal apoptotic activity in early KO retinal tissues. In contrast, several VLC ceramides, many containing one or more double bonds, were increased in both retina and RCS (Figs. 4A, S7B), with elevated abundance relative to Cer16:0 (Fig. 4E), indicating a shift in the ceramide pool toward VLC species.

Most strikingly, the normally rare, saturated VLC ceramide Cer26:0 was significantly enriched in the RCS of the combined cohort and females, with a similar upward trend in males (Fig. 4D). Its abundance relative to Cer16:0 was also increased (Fig. 4E), further supporting a shift of the ceramide pool toward VLC species. Accumulation of excess Cer26:0, which promotes membrane rigidity and is highly resistant to degradation [32, 33], is a pathological hallmark of X-linked adrenoleukodystrophy caused by ABCD1 mutations [34]. Its accumulation aligns with the upregulation of *Elovl1* and *Cers2* observed in CLIC4-KO RPE, as well as the downregulation of *Abcd1*, pointing to concurrent increases in VLC ceramide elongation and synthesis and reduced peroxisomal degradation.

Concurrently, female KO RCS also displayed a significant increase in the relative abundance of free cholesterol in CLIC4-KO RPE (Fig. 4F, G). In RCS, 20-hydroxycholesterol showed a nonsignificant trend toward an increase, whereas several other oxysterol species examined, including 4β- and 7β-hydroxycholesterol, showed only modest or inconsistent alterations across sexes and tissues (Fig. S8A–B).

### scRNA-seq identifies metabolic repression in dysfunctional rods

**Fig. 5:**
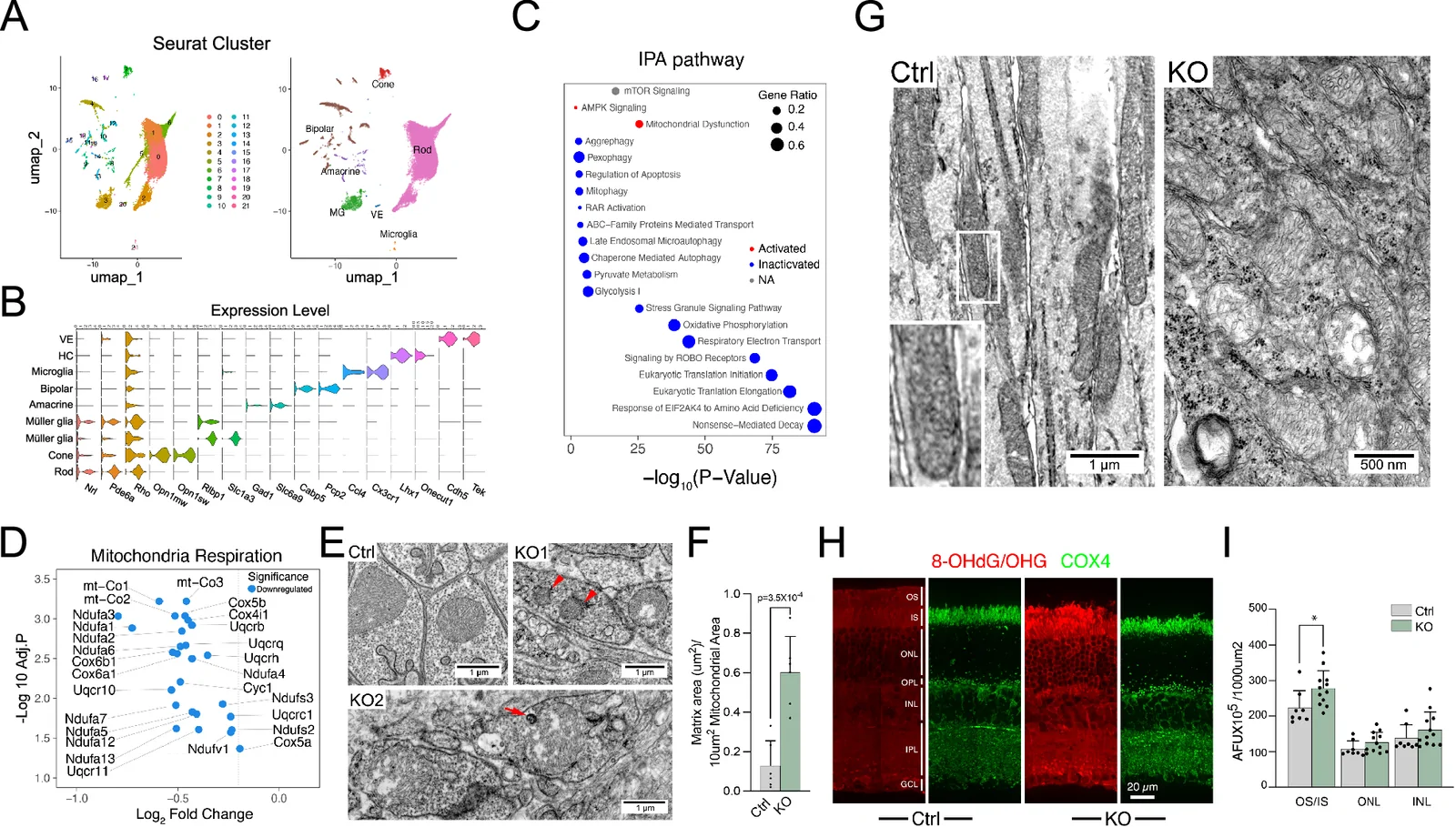
Single-cell transcriptomic and ultrastructural analysis reveals mitochondrial defects in rods of KO mice. **A** UMAP visualization of retinal scRNA-seq data with refined cell-type annotations. Clusters were initially identified using unsupervised graph-based clustering (left) and subsequently refined based on canonical marker gene expression and lineage-specific transcriptional programs (right). Each point represents a single cell and is colored by its final annotated cell type. In the right panel, clusters are labeled numerically based on shared nearest neighbor clustering at a resolution of 0.2 using the Seurat package. In the left panel, clusters are labeled by cell type. The initial analysis included four samples from two datasets, incorporating samples from other projects; only the two samples related to the CLIC4 project (KO and Ctrl) are shown here. See Figure S9 for evidence that the integration method effectively removed genotype-driven batch effects. **B** Violin plots of canonical marker gene expression used to annotate respective retinal cell types as indicated, confirming correct cell-type identification. HC: horizontal cells, VE: vascular endothelial cells. **C** Representative IPA-revealed canonical pathways overrepresented in IPA assay of DEGs in rods from KO versus Ctrl retinas. *P*-values were calculated using the Benjamini-Hochberg method for multiple testing. **D** Volcano plots showing fold changes (log₂ fold change (log₂FC) ≤ 0.2 and FDR-adjusted *P* values ≤ 0.05) of respiration complex genes affected in rods of KO (vs. Ctrl) retinas. **E**–**G** Electron micrographs of rod photoreceptors showing mitochondria in rod IN and synaptic terminals of 3-month-old Ctrl and KO mice. Mitochondria in KO, both in terminals (**E**) and IS (**G**), are characterized by swollen lumen with expanded empty space and reduced cristae. Red arrowheads point to two mitochondria within a single KO synaptic terminal. Red arrows denote electron-dense membranes at the boundary of the mitochondrial outer membrane. Images are representative of *n* = 3 female mice per genotype from two independent experiments. Quantification of mitochondrial matrix area in rod terminals is shown in (**F**). Data are presented as mean ± SD. A total of 34 mitochondria from Ctrl and 24 from KO mice were analyzed from three randomly selected electron micrograph fields per genotype (*N* = 2 mice per genotype). Matrix area (µm²) was normalized to 10 µm² of mitochondrial area for each mitochondrion measured. Statistical significance was determined using a two-tailed Student’s t-test; *p ≤ 0.05. **H** Representative retinal co-staining of 8-OHdG/8-OHG with Cox4 in 3-month-old mice. **I** Layer-stratified quantification of (**H**). Staining intensity (AUF) of 8-OHdG/8-OHG in the IS/OS, outer nuclear layer (ONL), and inner nuclear layer (INL) of retinas from Ctrl and KO mice. Data are presented as mean ± SD for *n* = 3 female mice per genotype, with 8 and 12 stained image fields quantified for the Ctrl and KO groups, respectively. Statistical significance was determined using a two-tailed Student’s t-test. *p ≤ 0.05. Source data of (**F**, **I**) are provided as a Source Data file.

To investigate transcriptional changes underlying early rod dysfunction caused by RPE-specific CLIC4 deficiency, we performed single-cell RNA sequencing (scRNA-seq) of retinas from 3-month-old KO and Ctrl mice, focusing on females. Integrated analysis resolved all major retinal cell types with minimal genotype-associated batch effects (Fig. S9). Unsupervised clustering identified 21 transcriptionally distinct populations, annotated using established cell type–specific markers (Fig. 5A, B). Rod cluster purity was confirmed bioinformatically by strong enrichment of rod signature genes (*Rho, Pde6a, Nrl*) and negligible expression of markers from other retinal cell types (Fig. 5B).

IPA of DEGs in KO versus control rods (Fig. 5C; Supplementary Table 3) revealed pronounced bioenergetic stress, characterized by predicted mitochondrial dysfunction and AMPK signaling activation. Multiple metabolic pathways, including glycolysis and pyruvate metabolism, were broadly suppressed. Pathways governing organelle quality control, including mitophagy, pexophagy, and autophagy, were also significantly reduced. Target gene analysis of DEGs further revealed coordinated suppression of mitochondrial ETC components across multiple complexes (Fig. 5D), consistent with mitochondrial dysfunction in KO rods.

IPA further revealed downregulation of proteostasis-related pathways, including translation initiation and elongation and nonsense-mediated mRNA decay. Cilium assembly and ABC transporter–mediated transport pathways were also suppressed (Fig. 5C). Given that the photoreceptor OS is a specialized cilium, suppression of cilium assembly pathways suggests impaired OS biogenesis. Among the downregulated ABC transporters, *Abca4*, a key mediator of photoreceptor retinoid trafficking, was reduced, pointing to potential impairment of all-trans-retinal clearance from the OS [28, 35, 36].

### Mitochondrial injury and oxidative damage in rods and RPE of RPE^*∆Clic4*^ Mice

Even modest lipid alterations can disrupt membrane curvature, cristae structure, and ETC organization, thereby impairing mitochondrial function [37–40]. To examine this in KO mice, we assessed mitochondrial structure in 3-month-old females using transmission electron microscopy. Rod photoreceptors have two anatomically distinct mitochondrial populations: a single large, round mitochondrion in the synaptic terminal, and elongated mitochondrial clusters longitudinally arrayed along the inner segment (IS) axis [41]. In KO rod terminals, mitochondria showed variable size and number, expanded matrix space, and electron-dense multilamellar structures (Fig. 5E, F). Mitochondria in IS were similarly swollen with reduced cristae density (Fig. 5G).

These structural abnormalities suggest compromised ETC function, which may trigger electron leak and superoxide generation. To examine this, we stained retinas with an 8-OHdG antibody, which recognizes oxidized guanine in both DNA (8-hydroxydeoxyguanosine, 8-OHdG) and RNA (8-hydroxyguanosine, 8-OHG). The 8-OHdG/8-OHG signal was significantly elevated across the photoreceptor IS and OS layers, but not in the outer or inner nuclear layers, in 3-month-old KO relative to Ctrl (Fig. 5H, I). The predominant cytoplasmic distribution suggests that the primary oxidative targets are cytoplasmic RNA or mitochondrial DNA and RNA, an interpretation further supported by the absence of nuclear staining.

Staining for 4-HNE, a marker of PUFA peroxidation and oxidative membrane damage, showed no difference between KO and Ctrl photoreceptor layers at 3 months (Fig. S10A, B), but became significantly elevated in the OS at 6 months in KO mice (Fig. S10C, D). Together, these findings suggest that mitochondrial structural injury and oxidative nucleic acid damage precede broader lipid peroxidation in rods, pointing to mitochondrial dysfunction as an early manifestation of photoreceptor stress secondary to CLIC4-deficient RPE.

## Discussion

Despite decades of evidence linking disrupted lipid homeostasis to dry AMD, the mechanisms by which lipid composition, abundance, and distribution become perturbed in stressed RPE, and how these perturbations propagate to impair rod function and drive disease progression remain poorly understood. Here, using a validated dry AMD mouse model in which RPE is primarily stressed by CLIC4 deficiency, we address this gap through early-stage multi-omic analysis.

### Integrated transcriptomic and lipidomic analyses reveal early mechanistic drivers of AMD

**Fig. 6:**
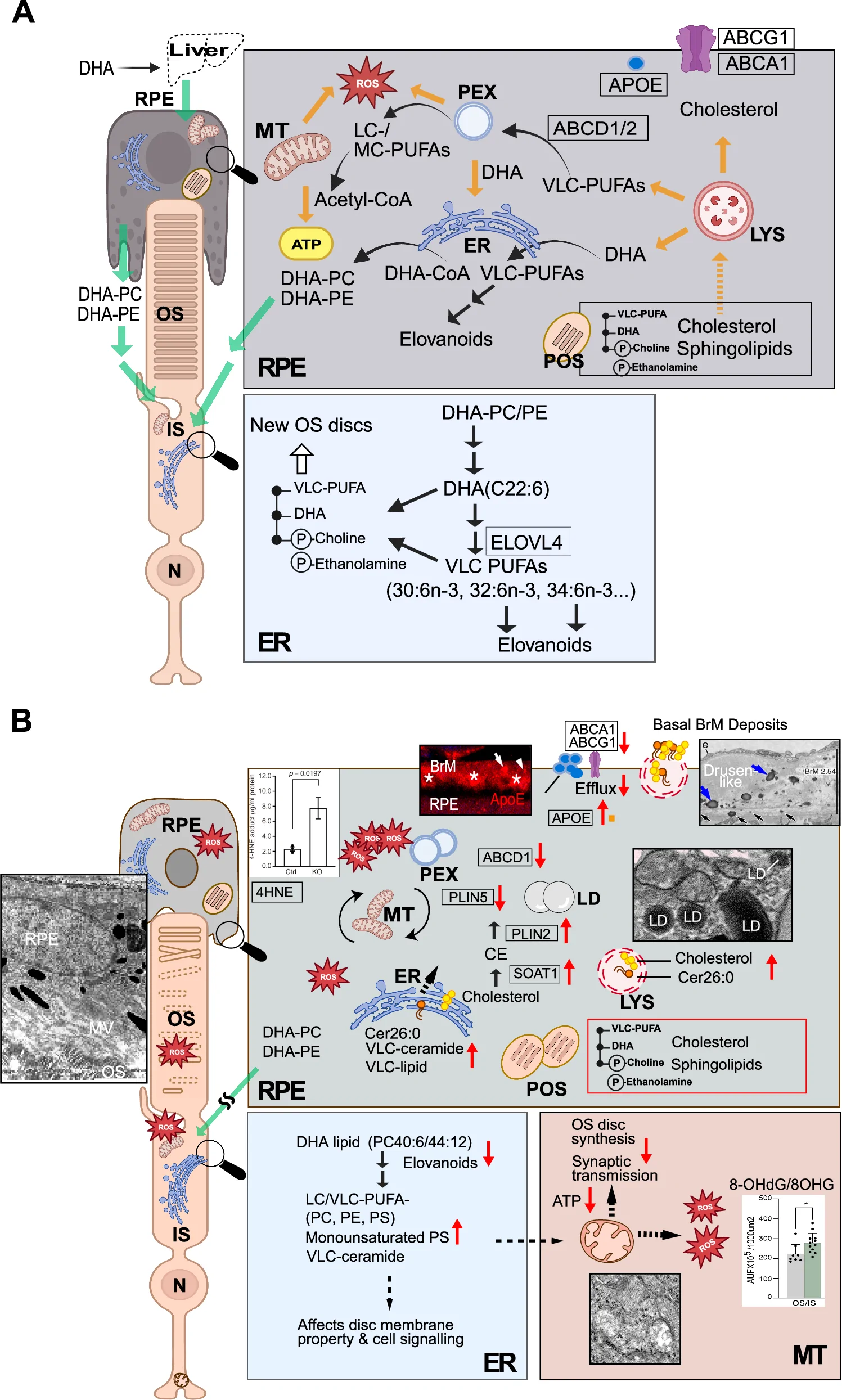
Outer retinal lipid homeostasis and its disruption in RPE-specific CLIC4 deficiency. **A** Steady-state lipid trafficking between photoreceptors and RPE. In photoreceptor IS, DHA from circulation or RPE recycling is activated to DHA-CoA in the ER and elongated by ELOVL4 into VLC-PUFAs, incorporated into DHA-phospholipids for OS disc assembly. Daily RPE phagocytosis of shed OS discs delivers lipid cargo to lysosomes, releasing DHA, VLC-PUFAs, and cholesterol. DHA is re-esterified into DHA-PC/PE for retrograde transfer to photoreceptors. VLC-PUFAs undergo peroxisomal β-oxidation via ABCD1/2, generating LC/MC-PUFAs for mitochondrial oxidation and ATP production, or are converted to elovanoids supporting membrane integrity. Excess cholesterol is effluxed basally via ABCA1-APOE, maintaining sub-RPE homeostasis. **B** Concurrently, ABCD1 downregulation reduces peroxisomal VLC-fatty acid import, expanding the cytosolic acyl-CoA pool available for de novo CerS2-mediated Cer26:0 synthesis in the ER. These PUFA-rich and VLC-ceramide species are highly peroxidation-prone, driving elevated 4-HNE adduct formation previously demonstrated by immunofluorescence in RPE^∆*Clic4*^ mice [19]. Lipidomic and transcriptomic evidence of increased cholesterol, increased SOAT1 promoting cholesterol esterification to cholesterol esters (CE), increased PLIN2 stabilizing LD surfaces, and downregulated PLIN5 suppressing LD-to-mitochondria lipid transfer collectively shift metabolism toward lipid storage at the expense of energy production. Reduced ABCA1/ABCG1 further limits basolateral cholesterol efflux; compensatory mTOR inhibition and PPARGC1α activation reflect an attempted mitochondria turnover but insufficient bioenergetic adaptation. The net result is secretion of APOE-laden, lipid-rich material into Bruch’s membrane (BrM), forming oil-rich drusen-like deposits demonstrated by APOE immunolabeling and EM [19]. Sub-RPE accumulation of cholesterol, Ce, Cer26:0 and VLC-ceramide is predicted to scaffold drusen nucleation and growth. Loss of RPE microvilli disrupts RPE–OS interdigitation and bidirectional lipid exchange. In rods, lipidomic remodeling—including relative depletion of canonical DHA-containing phospholipids and accumulation of monounsaturated phosphatidylserine and VLC ceramides—is predicted to alter membrane biophysics and mitochondrial function. Converging transcriptomic suppression of ETC components, ultrastructural mitochondrial abnormalities, and elevated 8-OHdG/8-OHG collectively support early mitochondrial dysfunction and rising oxidative damage. Transcriptomic and ultrastructural evidence of mitochondrial compromise is consistent with predicted impairment of energy-intensive rod processes—including rhodopsin phosphorylation, OS disc formation, and synaptic transmission—potentially contributing to the scotopic ERG decline observed in KO mice [19]. Temporally, mitochondria-derived oxidative nucleic acid damage precedes OS lipid peroxidation in rods, suggesting a progressive oxidative cascade from mitochondria to membranes. Together, cell-autonomous RPE lipid dysregulation and non-cell-autonomous photoreceptor failure establish a feed-forward cycle of outer retinal degeneration recapitulating hallmarks of dry AMD.

This study employed standard targeted shotgun lipidomic profiling to assess relative compositional changes within and across lipid classes [42] rather than absolute quantification, which represents a limitation of the current study. However, biologically active lipid composition is highly organized within subcellular membranes and organelles, and even subtle alterations in specific lipid species can substantially influence membrane biophysical properties, protein–lipid interactions, and organelle function, thereby exerting disproportionate effects on cellular integrity [43]. Here we found that lipid remodeling is an early and primary consequence of *Clic4* deficiency in RPE and is associated with coordinated transcriptional changes in pathways regulating lipid uptake, degradation, synthesis, and trafficking. A working model depicting lipid dyshomeostasis in the outer retinas of RPE^∆Clic4^ mice is shown in Fig. 6. For example, the concurrent downregulation of *Cd36*, *Fabp4*, and *Cebpa* in CLIC4-KO RPE cells suggests a broad disruption of lipid homeostasis encompassing impaired lipid uptake, mobilization, and transcriptional regulation. Importantly, these changes were detected at 3 months of age, several months before overt morphological or clinical phenotypes, and specifically within RPE cells in which *Clic4* was selectively deleted.

The ceramide profile identified in CLIC4-KO RPE is particularly noteworthy. It is known that subtle compositional shifts in ceramide-enrich membrane domains may alter signaling platforms and displace cholesterol from lipid rafts, collectively triggering profound cellular dysfunction [31]. High-resolution MALDI imaging identified VLC ceramides within drusen from AMD donors, as well as in sub-RPE deposits formed by porcine RPE monolayers [5, 6, 44]. The marked shift toward increased Cer26:0 at the expense of Cer16:0 in the RCS tissue of RPE^∆*Clic4*^ mice is remarkable, given that Cer26:0 is a saturated VLC ceramide that is normally rare in young control mice. Unlike shorter-chain ceramides, Cer26:0 preferentially partitions into highly ordered gel-phase membrane domains, conferring exceptional rigidity and resistance to both enzymatic hydrolysis and lysosomal degradation [32, 33]. Its accumulation, potentially driven by enhanced de novo synthesis (consistent with upregulation of *Elovl1* and *CerS2*) and/or impaired lysosomal clearance (consistent with downregulation of *Pla2g15* and autophagy-related pathways).

Free cholesterol accumulation in female KO RCS tissue is consistent with transcriptional upregulation of cholesterol biosynthetic genes (*Sqle*, *Srebf2*, *Soat1*) together with downregulation of cholesterol efflux transporters (*Abca1* and *Abcg1*). Transcriptomic analyses further support a model in which CLIC4 loss promotes LD accumulation through increased cholesterol esterification (elevated *Soat1*) and expansion of stable, storage-type LDs resistant to lipolytic turnover (increased *Plin2*, decreased *Plin5*). Although these findings strongly associate transcriptional dysregulation with lipid remodeling, definitive demonstration of causality will require intervention-based studies.

### Sex as a biological modifier of retinal lipid homeostasis in AMD

While the mixed-sex cohort demonstrates a shift toward longer-chain PUFA-containing PC, PE, and PS species, sex-stratified analyses reveal a divergent remodeling pattern in DHA-containing lipids. Female KO animals exhibit a redistribution of DHA-containing lipids between retina and RCS that is not apparent in males. Additionally, cholesterol accumulation in RCS is selectively observed in female KO animals, suggesting that sex may modulate sterol handling. These observations align with well-established sex differences in HDL, LDL, and apolipoprotein metabolism [45, 46], as well as reported reductions in elovanoid precursors in female AMD retinas [47]. Taken together, and in the context of the disproportionate burden of AMD in women ( ~ 65% of cases) not fully explained by longevity [48], these findings raise the possibility that sex-specific lipid trafficking contributes to differential disease vulnerability.

Whether this sex-dependent remodeling reflects altered sterol transport, PUFA retention, or differential biosynthetic flux remains to be determined, and will require larger cohorts powered to detect sex-by-genotype interactions and absolute lipid quantification. The absence of sex-stratified transcriptomic analysis represents an additional limitation and an important direction for future work.

### RPE lipid dyshomeostasis and oxidative stress: implications for Dry AMD

Our data support a model in which RPE-specific CLIC4 deficiency–driven lipid dyshomeostasis contributes to AMD pathology through several converging mechanisms. First, the lipid raft–rich apical microvilli and basal infoldings of the RPE are critical structural platforms for photoreceptor outer segment lipid recycling and choroidal lipid efflux, respectively. The shifts toward increased phospholipid unsaturation and accumulation of longer-chain ceramides in CLIC4-deficient RPE may compromise membrane fluidity, curvature, and structural integrity of these specialized domains, consistent with the marked loss of microvilli and basal infoldings observed in 3-month-old KO mice [19], which would be expected to severely impair bidirectional lipid exchange at the photoreceptor-RPE-choroid interface. The reciprocal shift of DHA-containing lipids, i.e., enriched in RCS and relatively reduced in neural retina, is consistent with impaired vectorial lipid trafficking rather than a primary biosynthetic defect. This pattern suggests that CLIC4 modulates VLC-PUFA compartmentalization primarily through spatial distribution across retinal compartments, a mechanism distinct from ACSL6 and LPLAT3, which enzymatically regulate VLC-PUFA biosynthesis and membrane incorporation [26, 49, 50]. Consistent with this distinction, RPE-specific CLIC4 deficiency does not recapitulate the marked photoreceptor degeneration characteristic of ACSL6-deficient mice [51].

Second, lipid remodeling observed in CLIC4-deficient RPE could carry direct consequences for oxidative stress. The relative enrichment of unsaturated phospholipids increases membrane oxidative vulnerability, consistent with elevated 4-HNE levels detected in KO RPE [19]. Ceramide accumulation has been linked to RPE inflammation and complement dysregulation [52, 53], and may contribute to the subRPE C3a immunoreactivity observed in the KO mice [19].

Third, free cholesterol accumulation in female KO RCS, together with transcriptionally upregulated cholesterol esterification enzymes and PLIN2/3 may contribute to intracellular LD expansion and extracellular lipid deposition at Bruch’s membrane [19]. The concurrent shift toward saturated Cer26:0, which promotes rigid, degradation-resistant membrane domains, may further favor the formation of drusen-like sub-RPE deposits observed in KO mice [19] and is consistent with VLC ceramide–enriched deposits identified in drusen from AMD donors. It remains unclear whether the coordinated shift in lipid composition, or a few dominant toxic species, is sufficient to initiate AMD-associated pathology. We also acknowledge that direct causal relationships between lipid remodeling and AMD pathology will require targeted interventions to establish, which are the focus of future studies.

### Early rod mitochondrial dysfunction in CLIC4-deficient retina: a potential biomarker of dry AMD onset

Notably, CLIC4-deficient RPE and neighboring rod photoreceptors mount divergent cellular responses to lipid dyshomeostasis. RPE activates compensatory responses by promoting clearance and biogenesis of damaged organelles including mitochondria, whereas rods exhibit transcriptomic suppression of bioenergetic and organelle-turnover pathways. Mitochondrial dysfunction in rods is supported by converging transcriptomic, ultrastructural, and oxidative stress evidence. Temporally, rod IS and OS show selective elevation of 8-OHdG/8-OHG before detectable 4-HNE accumulation, suggesting that mitochondria-derived oxidative nucleic acid damage precedes broader lipid peroxidation in rods.

Mitochondrial abnormalities in rods predict limitations in energy supply for rhodopsin phosphorylation, outer segment disc formation, and sustained glutamate release at ribbon synapses, the latter depending critically on synaptic terminal mitochondria for ATP production. Damaged mitochondria observed in rod terminals may therefore contribute to the delayed dark adaptation and ERG decline observed in KO mice [19].

Photoreceptors and RPE maintain a tightly coordinated metabolic ecosystem through bidirectional carbon-energy exchange [54], whereby rod photoreceptors convert glucose to lactate to fuel RPE oxidative metabolism, while RPE conserves glucose for photoreceptors. Perturbations of this coupling have been proposed to contribute to AMD pathogenesis [55]. We propose that impaired mitochondria in rods of RPE^∆*Clic4*^ may compromise NAD⁺/NADH homeostasis required to sustain glycolytic flux, potentially reducing lactate export to RPE and attenuating carbon-energy coupling. It is further plausible that oxidatively damaged OS arriving at already-stressed RPE via daily phagocytosis exacerbate RPE metabolic burden. These two pathogenic axes—lipid dyshomeostasis and attenuated carbon-energy coupling—reinforce a feed-forward cycle of outer retinal injury. Delineating metabolic flux in RPE and rods will be important to further understand the temporal interaction between these pathogenic pathways.

These findings carry direct translational relevance: early diagnosis of dry AMD remains an unmet clinical need. Current clinical diagnosis relies on fundus drusen detection, yet by this stage cellular stress and photoreceptor damage have already begun, and early detection of retinal changes before drusen appear remains an unmet need. While delayed dark adaptation can predict dry AMD years before drusen become clinically apparent [1], its widespread adoption remains limited by testing complexity and duration. Emerging spectral-domain optical coherence tomography studies report progressive decline in ellipsoid zone reflectivity, corresponding to the mitochondria-rich IS/OS junction, with advancing dry AMD [56, 57]. Together, these observations position rod mitochondrial structural integrity as a candidate imaging biomarker for pre-symptomatic dry AMD detection, warranting future investigation in human cohorts.

In summary, our findings identify RPE-driven lipid dysregulation as an early initiator of interconnected AMD pathologies, linking disrupted lipid recycling, oxidative stress, mitochondrial dysfunction, and drusen formation into a coherent disease framework. The RPE^∆*Clic4*^ mouse model provides a tractable platform for studying these initiating events and testing mechanism-based therapies.

## Materials and methods

### Mice

Mice were maintained on a standard 12:12 h light: dark cycle in a vivarium at 21.5 ± 1 °C with 30–70% relative humidity and provided ad libitum access to standard chow (LabDiet 5053, PMI, St. Louis, MO). As previously described [19], Best1-Cre⁺/⁻; *Clic4*^f/f^ males were crossed with *Clic4*^f/f^ females to generate Best1-Cre⁺/⁻;*Clic*4^f/f^ (RPE^∆*Clic4*^, KO) mice and littermate Best1-Cre⁻/⁻;*Clic4*^f/f^ Ctrl mice. The RPE-specific Cre expression of Best1-Cre^+/−^ mice was reported to start postnatal 10–15 day-old [58]. RPE-specific *Clic4* loss was confirmed by staining and RNAseq [19]. All mice were genotyped and confirmed to be negative for the retinal degeneration 8 (rd8) allele. Animal ages and sample sizes are provided in the figure legends.

### Lipid extraction and LC-MS/MS-based lipidomic analysis

For lipidomic assays, the neural retina was carefully peeled from the RCS. Both tissues were snap-frozen (*n* = 3 per gender, per genotype) and stored at −80 °C until analysis. The purity of the retinal samples was confirmed by high expression of rhodopsin and minimal contamination, as indicated by qPCR analysis of *Rpe65* and *Plvap* (see Fig. 4A in [19]).

Targeted LC-MS/MS lipidomic analyses and lipid extraction procedures were performed as previously described [59]. Briefly, each sample was homogenized in methanol (3 ml) and CHCl₃ (6 ml) containing an internal standard mixture (AA-d8, PGD2-d4, EPA-d5, 15-HETE-d8, LTB4-d4, and PC(28:0)), sonicated in an ice-water bath, and stored at −80 °C overnight. After centrifugation, the pellet was washed with CHCl₃/MeOH (2:1), and the supernatants were combined. Distilled water (pH 3.5) was added, aqueous and organic phases separated, and the lower organic phase was dried under N₂. The dried lipid extracts were resuspended in methyl formate, dried again, and reconstituted in MeOH/H₂O for analysis. Base hydrolysis was used to release total fatty acids from complex lipid extracts. Samples were subjected to alkaline hydrolysis using sodium hydroxide (NaOH) to cleave esterified fatty acids from phospholipids, neutral lipids, and other lipid classes. This approach enables efficient recovery of total fatty acids for downstream analysis, as previously described [60].To determine total DHA, AA, and their derivatives, samples were hydrolyzed with NaOH at 42 °C for 3 h, extracted with ethyl acetate, dried, and resuspended in MeOH/H₂O (2:1).

LC-MS/MS analysis of VLC-PUFAs was performed on a Xevo TQ-S coupled with an Acquity I-Class UPLC (Waters) using an Acquity UPLC HSS T3 column (1.8 µm, 2.1 × 50 mm). Solvent A (H₂O + 0.1% acetic acid) and solvent B (90% acetonitrile, 10% isopropanol) were run at 0.6 ml/min with a gradient from 25% to 100% B over 7.5 min, held for 2.5 min, then re-equilibrated for 2 min. Capillary voltage was −2.5 kV; desolvation temperature, 600 °C; desolvation gas, 1100 L/h; cone gas, 150 L/h; nebulizer pressure, 7.0 bar; source temperature, 150 °C. Details on how VLC-PUFAs were identified and quantified can be found in the supporting information (Table S4).

For phospholipid molecular species analysis, an Acquity UPLC BEH Amide column (1.7 µm, 2.1 × 100 mm) was used with solvent A (acetonitrile:water 1:1, 10 mM ammonium acetate, pH 9.1) and solvent B (acetonitrile:water 95:5, 10 mM ammonium acetate, pH 9.1). Solvent B (100%) was run isocratically for 5 min, then linearly transitioned to 20% A over 8 min, followed by 65% A for 0.5 min, held isocratically at 65% A for 3 min, and returned to 100% B over 3.5 min for re-equilibration. The capillary voltage was 2.5 kV; desolvation temperature, 550 °C; desolvation gas, 800 L/h; cone gas, 150 L/h; nebulizer pressure, 7 bar; and source temperature, 120 °C. Semi‑quantitative analysis of phospholipids was performed by integrating peak areas from the parent‑ion scanning function and normalizing to the internal standard PC 28:0. A description of how phospholipid species were identified and quantified can be found in the Supplementary Information (Table S5)

Lipid standards (Cayman Chemical, Ann Arbor, MI) were used for instrument tuning, optimization, and calibration curve generation. Each sample was analyzed at least four times, and concentrations were calculated using both calibration curves and a one-point comparison method. The typical ratio of unesterified to total endogenous DHA is >10,000, which was used as a quality criterion to ensure proper tissue handling and accurate extraction and measurement.

### Bulk RNA-seq and bioinformatic analysis of purified RPE cells

We analyzed previously published bulk RNA-seq data from enzymatically purified RPE cells isolated from the retinas and choroid/sclera cups of 3-month-old KO and Ctrl mice [19]. RPE purity was confirmed by enrichment of Rpe65 mRNA and minimal contamination by retinal (*Rho*) and choroidal (*Plvap*, plasmalemma vesicle-associated protein) marker transcripts, as assessed by qPCR (see Supplementary Fig. 4a in [19]). DEGs reported in that study were used to calculate log₂ fold changes and false discovery rate (FDR)-adjusted p-values for downstream analyses. Canonical pathway enrichment and upstream regulator analyses were performed using IPA (QIAGEN Bioinformatics).

### scRNA seq and bioinformatic analysis of dissociated rods

Four retinas from 3-month-old female KO and Ctrl mice were pooled in ice-cold EBSS and incubated with 1 ml of a solution containing papain (20 U/ml) and DNaseI in EBSS for 40 min at 25 °C with gentle shaking every 10 min. Enzymatic digestion was stopped by adding 300 µl of ovomucoid solution, followed by an additional 150 µl of DNase I. The tissue was gently dissociated by trituration using a 5 ml pipette. An equal volume of HBSS containing 1.5% BSA was added to the cell suspension, which was then filtered through a sterile 70 µm mesh and centrifuged at 300 g for 5 min. The supernatant was discarded, and the cell pellet was gently resuspended in PBS (without Ca²⁺ and Mg²⁺) containing 0.04% BSA to a final concentration of 1 × 10⁶ cells/ml. Single-cell libraries were prepared following the 10x Genomics Chromium Single Cell 3′ Reagent Kits v4 User Guide (CG000731) using GEM-X chemistry. A total of 29,000 cells were loaded onto the Chromium Controller, and libraries were sequenced to a depth of 20,000 reads per cell on an Illumina NovaSeq X Plus platform.

Sequencing data were mapped to the GRCm38.p5 genome assembly and were converted to a cell-gene matrix with the Cell Ranger pipeline. Downstream analysis was performed with. All analyses were carried out on NIH biowulf2 high performance computing environment. We used the following R packages for the analyses: Seurat_5.3.0, DESeq2_1.48.1, fgsea_1.34.2, and msigdbr_25.1.1. The Seurat package [61] was used for clustering and UMAP analysis. Differential gene expression between knockout and wildtype was performed using DESeq2 [62]. Clusters mapping to cell types, such as rod cells, cone cells, etc., were based on custom-curated gene signatures. Statistics from the DESeq2 analysis as rank list were used to perform GSEA analysis (FDR < 0.01) using the Hallmark gene sets from MSigDB (https://www.gsea-msigdb.org/gsea/msigdb/index.jsp).

Retina cell clusters were manually annotated based on classical marker genes [63], and differential gene expression analysis between KO and Ctrl cells was performed using the limma package [64]. The limma algorithm computed the log₂ fold change (log₂FC) and FDR-adjusted *P* values for each gene. Multiple testing correction was conducted using the Benjamini–Hochberg method. Genes with an adjusted *P* value ≤ 0.050 and |log₂FC | >0.2 were defined as DEGs. Heatmaps were generated using the heatmap package in R (v4.3.3). Dot plots were generated using the DotPlot function in Seurat. Heatmaps were generated using the heatmap package to visualize the gene expression fold-change values (logFC). The gene–logFC pairs were converted into numeric matrices, where rows corresponded to individual genes and columns represented log fold-change values. All transformations were A fixed color scale ranging from –2 to +2 was applied.

### Retinal immunohistochemistry, imaging, and quantification

For immunostaining, eyecups were immersion-fixed in 4% paraformaldehyde (PFA) for approximately 1 hour at room temperature. Fixed eyecups were embedded in agarose II (Fisher Scientific) and sectioned at 40 μm using a vibratome. Sections were processed using a free-floating immunostaining protocol. The following primary antibodies were used: anti-8-OHdG/8-OHG goat antibody (AB5830, Millipore, 1:500; RRID: AB_10805953), anti-4-HNE rabbit antibody (Alpha Diagnostics, HNE1-S, 1:500; RRID: AB_2629286), and anti-CoxIV mouse antibody (BioLegend, 937801, 1:500; RRID: AB_2565611). Alexa Fluor-conjugated secondary antibodies were obtained from Thermo Fisher Scientific. Confocal images were acquired using a Zeiss spinning disc Observer Z1 confocal microscope. Autofluorescence emission spectra were characterized by lambda scanning on a Leica Stellaris 8 confocal microscope to determine peak fluorescence wavelengths. Images were processed for presentation using Adobe Photoshop (v25.9) and Adobe Illustrator (v28.5).

For quantification, images acquired in CZI format were imported into ImageJ. Regions of interest (ROIs) were manually outlined using the freehand selection tool and saved in the ROI Manager. Fluorescence intensity within each ROI was measured using the Analyze function, capturing area and integrated density. Staining intensity was normalized to fluorescence per 1000 μm².

### Transmission EM and quantification of mitochondrial structural change

Three-month-old KO and Ctrl female mice were transcardially perfused with heparinized saline (20 U/ml), followed by 4% PFA/3.75% acrolein and then 4% PFA in 0.1 M phosphate buffer (pH 7.4). Eyes were enucleated, eyecups prepared, and tissues post-fixed in 2.5% glutaraldehyde in 0.1 M cacodylate buffer at 4 °C. Small eyecup pieces ( ~ 2 × 2 mm) were sectioned at 120 µm using a vibratome and processed for en bloc staining, as described [65]. After washes in cacodylate buffer containing 2 mM CaCl₂, sections were treated with potassium ferrocyanide/osmium tetroxide, thiocarbohydrazide, and a second osmium tetroxide fixation. Samples were dehydrated through graded ethanol, embedded in Epon, and ultrathin sections (72 nm) were collected on G400-Cu grids and examined using a Philips CM10 transmission electron microscope.

Quantification of rod terminal mitochondrial matrix area was done using ImageJ, as described [66, 67]. EM images with a minimum of 3 rod terminals were randomly selected. After importing image in ImageJ, each mitochondrion was traced using the free hand tool and area determined by selecting ‘Analyze’-‘Measure’. Keeping the tracing of the mitochondria in place, the image threshold was adjusted to cover the matrix area and determined once again by selecting ‘Analyze’-‘Measure’. This was repeated for every rod terminal mitochondrion in the selected image. The matrix area obtained was back calculated for every 10 µm² mitochondria area.

### Statistics

Statistical analyses were performed using GraphPad Prism (versions 6 or 8) or Microsoft Excel for Mac (Version 16.16.27). Data are presented as mean ± SD unless otherwise indicated. Normality was assessed using the Shapiro–Wilk test. For comparisons between two groups, an unpaired two-tailed Student’s t-test was used for normally distributed data, while the Mann–Whitney test was applied for non-normally distributed data. The number of experimental replicates and the specific statistical tests used are indicated in the figure legends. Differences were considered statistically significant at *P* ≤ 0.05.

## Resource availability

Requests for further information and resources should be directed to and will be fulfilled by the lead contact, Ching-Hwa Sung (chsung@med.cornell.edu).

## Materials availability

All data are available in the main text or the supplementary materials. All R scripts and processed data supporting the findings of this study are available from the corresponding author upon reasonable request.

## Supplementary information

Supplementary Information

Figure S1

Figure S2

Figure S3

Figure S4

Figure S5

Figure S6

Figure S7

Figure S8

Figure S9

Figure S10

Table S1

Table S2

Table S3

Table S4

Table S5

Data source file

## Data Availability

All data are available in the main text or the supplementary materials. All R scripts and processed data supporting the findings of this study are available from the corresponding author upon reasonable request.

## Code availability

All data are available in the main text or the supplementary materials. All R scripts and processed data supporting the findings of this study are available from the corresponding author upon reasonable request.
