# Supplementary material for: Lipidomic and transcriptomic remodeling underlies early retinal pathogenesis in dry age-related macular degeneration
Source: Cell Death Dis. 2026 Aug 7;17(1):937. doi: 10.1038/s41419-026-09147-5 (PMC13736989; doi:10.1038/s41419-026-09147-5)
Supplement: Supplementary file 1 [file 41419_2026_9147_MOESM12_ESM.docx]

| **Acyl Chain Remodeling / Phospholipid Metabolism** | **Sterol Transport / Sphingolipid Metabolism** | **USFA Synthesis** | **Master Regulators** | **Mitochondria Homeostasis** | **Peroxisomes** | **Mitochondrial Respiration** |
| --- | --- | --- | --- | --- | --- | --- |
| Abca1 | Abca1 | Abcd1 | Abhd5 | Bnip3 | Abcd1 | Atp5f1a |
| Abca7 | Abcg1 | Acaa1a | Abhd5 | Bnip3l | Abcd2 | Atp5f1b |
| Acsl1 | Abcg4 | Acaa1b | Cdk1 | Calcoco2 | Abcd3 | Atp5mc1 |
| Acsl6 | Abcg5 | Acot1 | Cebpa | Dnm1l | Acaa1 | Atp5me |
| Bin1 | Abcg8 | Acot11 | Crebbp | Fis1 | Acox1 | Atp5pb |
| Cpt2 | Acer1 | Acot13 | Dagla | Fundc1 | Acox2 | Cox4i1 |
| Fig4 | Acer2 | Acot2 | Daglb | Gdap1 | Agps | Cox5a |
| Inpp5b | Acer3 | Acot4 | Dgat1 | Mff | Amacr | Cox5b |
| Inpp5d | ApoE | Acot7 | Dgat2 | Mfn1 | Cat | Cox6a1 |
| Inpp5e | Arsa | Acot8 | Egr1 | Mfn2 | Ech1 | Cox6b1 |
| Inppl1 | Asah1 | Agpat1 | Fabp4 | Mief1 | Far1 | Cyc1 |
| Lpcat1 | Asah2 | Baat | Foxo1 | Mief2 | Far2 | mt-Co1 |
| Lpcat2 | B3galt4 | Cd36 | G0s2 | Nfe2l2 | Gnpat | mt-Co2 |
| Lpcat3 | B4galt5 | Elovl1 | Hilpda | Nrf1 | Hsd17b4 | mt-Co3 |
| Lpcat4 | B4galt6 | Elovl2 | Lamp2 | Opa1 | Pex1 | Ndufa1 |
| Mfsd2a | B4galt7 | Elovl3 | Lipa | Optn | Pex10 | Ndufa10 |
| Ocrl | Cerk | Elovl4 | Lipe | Park2 | Pex11a | Ndufa11 |
| Picalm | Cerkl | Elovl5 | Lipe | Pink1 | Pex11b | Ndufa12 |
| Pik3c2a | Cers1 | Elovl6 | Lipe | Polg | Pex11g | Ndufa13 |
| Pik3c2b | Cers2 | Elovl7 | Mapk1 | Polg2 | Pex12 | Ndufa2 |
| Pik3c2g | Cers3 | Hacd1 | Mapk2 | Ppargc1a | Pex13 | Ndufa3 |
| Pla2g10 | Cers4 | Hacd2 | Mapk3 | Ppargc1b | Pex14 | Ndufa4 |
| Pla2g12a | Cers5 | Hacd3 | Mapk6 | Sirt1 | Pex16 | Ndufa5 |
| Pla2g12b | Cers6 | Hacd4 | Mgll | Sirt3 | Pex19 | Ndufa6 |
| Pla2g15 | Ch25h | Scd1 | Mtor | Sqstm1 | Pex2 | Ndufa7 |
| Pla2g16 | Cyp27a1 | Scd2 | Nr1h2 | Tfam | Pex26 | Ndufa8 |
| Pla2g2a | Degs1 | Scd3 | Nr1h3 | Tfb1m | Pex3 | Ndufa9 |
| Pla2g2c | Degs2 | Scd4 | Nr5a1 | Tfb2m | Pex5 | Ndufaf1 |
| Pla2g2d | Gal3st1 | Vldlr | Plin1 | Tfb2m | Pex6 | Ndufs1 |
| Pla2g2e | Galc |  | Plin1 | Tomm20 | Pex7 | Ndufs2 |
| Pla2g2f | Gba |  | Plin2 |  | Prdx5 | Ndufs3 |
| Pla2g3 | Gba2 |  | Plin3 |  | Scp2 | Ndufv1 |
| Pla2g4a | Gla |  | Plin4 |  |  | Ndufv2 |
| Pla2g4b | Hmgcr |  | Plin5 |  |  | Sdha |
| Pla2g4c | Kdsr |  | Pnpla2 |  |  | Sdhb |
| Pla2g4d | Ldlr |  | Pnpla2 |  |  | Sdhc |
| Pla2g4e | Naaa |  | Pnpla8 |  |  | Sdhd |
| Pla2g4f | Neu1 |  | Ppara |  |  | Uqcr10 |
| Pla2g5 | Neu2 |  | Ppard |  |  | Uqcr11 |
| Pla2g6 | Neu3 |  | Pparg |  |  | Uqcrb |
| Pla2g7 | Neu4 |  | Ppargc1a |  |  | Uqcrc1 |
| Plcb1 | Npc1 |  | Ppargc1b |  |  | Uqcrc2 |
| Plcb2 | Npc1l1 |  | Prkaca |  |  | Uqcrh |
| Plcb3 | Npc2 |  | Prkacb |  |  | Uqcrq |
| Plcb4 | Psap |  | Prkg1 |  |  |  |
| Plcd1 | Scarb1 |  | Prkg2 |  |  |  |
| Plcd3 | Sgms1 |  | Rictor |  |  |  |
| Plcd4 | Sgms2 |  | Rptor |  |  |  |
| Plcg1 | Sgpl1 |  | Rxra |  |  |  |
| Plcg2 | Sgpp1 |  | Rxrb |  |  |  |
| Plch1 | Sgpp2 |  | Rxrg |  |  |  |
| Plch2 | Smpd1 |  | Sirt1 |  |  |  |
| Plcz1 | Smpd2 |  | Sirt2 |  |  |  |
| Pld1 | Smpd3 |  | Sirt3 |  |  |  |
| Pld2 | Smpd4 |  | Sirt4 |  |  |  |
| Pld3 | Soat1 |  | Sirt5 |  |  |  |
| Pld4 | Soat2 |  | Sirt6 |  |  |  |
| Pld5 | Sphk1 |  | Sirt7 |  |  |  |
| Pld6 | Sphk2 |  | Snai1 |  |  |  |
| Synj1 | Sptlc1 |  | Sp1 |  |  |  |
| Synj2 | Sptlc2 |  | Srebf1 |  |  |  |
| Vac14 | Sptlc3 |  | Srebf1 |  |  |  |
|  | SqleA |  | Srebf2 |  |  |  |
|  | St3gal5 |  | Srebf2 |  |  |  |
|  | St8sia1 |  | Stat5b |  |  |  |
|  | St8sia5 |  | Tfe3 |  |  |  |
|  |  |  | Ywhab |  |  |  |
|  |  |  | Ywhae |  |  |  |
|  |  |  | Ywhaz |  |  |  |
