# Supplementary material for: Lipidomic and transcriptomic remodeling underlies early retinal pathogenesis in dry age-related macular degeneration
Source: Cell Death Dis. 2026 Aug 7;17(1):937. doi: 10.1038/s41419-026-09147-5 (PMC13736989; doi:10.1038/s41419-026-09147-5)
Supplement: Supplementary file 2 [file 41419_2026_9147_MOESM13_ESM.docx]

**Table S2. Ingenuity Pathway Analysis (IPA) — Canonical Pathways**

| **Ingenuity Canonical Pathways** | **-log(p-value)** | **Ratio** | **z-score** | **Molecules** |
| --- | --- | --- | --- | --- |
| **Mitochondrial Dysfunction** | 22.80 | 0.596 | N/A | ACO1, ACO2, AIFM1, Aph1c, APP, Atp5e, ATP5F1A, ATP5F1B, ATP5F1C, ATP5F1D, ATP5MC1, ATP5MC3, ATP5MF, ATP5MG, ATP5PB, ATP5PD, ATP5PF, ATP5PO, BACE1, BACE2, CAT, COX15, COX4I1, COX5A, COX6A1, COX6B1, Cox6c, COX7A1, COX7A2, COX7B, COX8A, Cox8b, CPT1A, CPT1B, CYB5A, CYC1, CYCS, GPX4, HSD17B10, HTRA2, LRRK2, MAOB, MAPK12, NDUFA1, NDUFA10, NDUFA11, NDUFA12, NDUFA13, NDUFA2, NDUFA3, NDUFA4, NDUFA4L2, NDUFA5, NDUFA6, NDUFA7, NDUFA8, NDUFA9, NDUFAB1, NDUFAF2, NDUFB10, NDUFB11, NDUFB3, NDUFB4, NDUFB5, NDUFB6, NDUFB7, NDUFB8, NDUFB9, NDUFS1, NDUFS2, NDUFS3, NDUFS4, NDUFS6, NDUFS7, NDUFS8, NDUFV1, NDUFV2, NDUFV3, OGDH, PARK7, PDHA1, PINK1, PRDX5, PSENEN, RHOT2, SDHA, SDHB, SDHC, SDHD, SOD2, TXN2, UCP2, UQCR10, UQCR11, UQCRB, UQCRC1, UQCRC2, UQCRFS1, UQCRQ, VDAC1, VDAC2, XDH |
| **Oxidative Phosphorylation** | 19.20 | 0.651 | 8.426 | Atp5e, ATP5F1A, ATP5F1B, ATP5F1C, ATP5F1D, ATP5MC1, ATP5MC3, ATP5MF, ATP5MG, ATP5PB, ATP5PD, ATP5PF, ATP5PO, COX15, COX4I1, COX5A, COX6A1, COX6B1, Cox6c, COX7A1, COX7A2, COX7B, COX8A, Cox8b, CYB5A, CYC1, CYCS, NDUFA1, NDUFA10, NDUFA11, NDUFA12, NDUFA13, NDUFA2, NDUFA3, NDUFA4, NDUFA5, NDUFA6, NDUFA7, NDUFA8, NDUFA9, NDUFAB1, NDUFB10, NDUFB11, NDUFB3, NDUFB4, NDUFB5, NDUFB6, NDUFB7, NDUFB8, NDUFB9, NDUFS1, NDUFS2, NDUFS3, NDUFS4, NDUFS6, NDUFS7, NDUFS8, NDUFV1, NDUFV2, NDUFV3, SDHA, SDHB, SDHC, SDHD, UQCR10, UQCR11, UQCRB, UQCRC1, UQCRC2, UQCRFS1, UQCRQ |
| **PPARα/RXRα Activation** | 11.40 | 0.468 | 3.204 | ACAA1, ACADL, ACOX1, ACVR1, ACVR1B, ADCY4, ADCY5, ADCY7, ADIPOR1, ADIPOR2, AP2A2, BCL3, BMPR2, CD36, CHUK, CKAP5, CPT1B, Cyp2c23, GHR, GNA14, GNAQ, GOT2, HELZ2, HSP90AA1, HSP90B1, IKBKB, IKBKE, IL1B, IL1R1, IL1RAP, IL1RL1, IL6, IRS1, ITGB5, JAK2, JUN, KRAS, LPL, MAP2K1, MAP2K2, MAP2K3, MAP3K14, MAP4K4, MAPK14, MEF2C, NCOA3, NCOR2, NFKB1, NFKB2, NFKBIA, NFKBID, NFKBIE, NR2F1, PDIA3, PLCB1, PLCB2, PLCB3, PLCD1, PLCD3, PLCE1, PLCG2, PLCL1, PLCL2, PPARGC1A, PRKAA2, PRKAB1, PRKAB2, PRKACA, PRKACB, PRKAR1B, PRKAR2B, PRKCA, PRKCB, RAP1B, RAP2A, RAP2B, RASD1, RELA, RRAS, RXRA, SHC1, SLC27A1, SMAD3, SMAD4, TGFB1, TGFB2, TGFB3, TGFBR1, TGFBR2 |
| **p70S6K Signaling** | 8.74 | 0.481 | -4.202 | AGTR1, AKT1, BTK, CD19, CD79A, CD79B, EEF2, EEF2K, EGFR, F2R, GNAI2, GNAQ, IL2RG, IL4R, IRS1, JAK1, KRAS, LYN, MAP2K1, MAP2K2, MTOR, PDIA3, PIK3C2B, PIK3CB, PIK3CD, PIK3CG, PIK3R5, PIK3R6, PLCB1, PLCB2, PLCB3, PLCD1, PLCD3, PLCE1, PLCG2, PLCL1, PLCL2, PLD1, PPP2CA, PPP2R1A, PPP2R2C, PRKCA, PRKCB, PRKCD, PRKCG, PRKCI, PRKCQ, PRKCZ, PRKD1, PRKD3, PTPA, RAP1B, RAP2A, RAP2B, RASD1, RRAS, SHC1, SRC, SYK, YWHAB, YWHAE, YWHAH |
| **mTOR Signaling** | 6.84 | 0.400 | -3.328 | AKT1, ARHGAP8/PRR5-ARHGAP8, DDIT4, DGKZ, EIF3C, EIF3K, EIF4EBP1, EIF4G1, EIF4G2, EIF4G3, FAU, GPLD1, HIF1A, IRS1, KRAS, MTOR, NAPEPLD, PGF, PIK3C2B, PIK3CB, PIK3CD, PIK3CG, PIK3R5, PIK3R6, PLD1, PLD2, PLD4, PLD5, PPP2CA, PPP2R1A, PPP2R2C, PRKAA2, PRKAB1, PRKAB2, PRKCA, PRKCB, PRKCD, PRKCG, PRKCI, PRKCQ, PRKCZ, PRKD1, PRKD3, PRR5, PTPA, RAC1, RAC2, RAP1B, RAP2A, RAP2B, RASD1, RHEB, RHOA, RHOB, RHOBTB2, RHOD, RHOH, RHOT2, RND1, RND3, RPS10, RPS11, RPS12, RPS19, RPS2, RPS20, RPS21, RPS23, RPS24, RPS27L, RPS3, RPS4Y1, RPS5, RPS6KA2, RPS6KA3, RPS6KA4, RPS6KB2, RPS8, RPSA, RRAS, ULK1, VEGFA, VEGFB, VEGFD |
| **Superpathway of Cholesterol Biosynthesis** | 2.97 | 0.517 | 3.742 | ACAA2, ACAT1, ACAT2, CYP51A1, DHCR24, FDFT1, HADHA, HADHB, HMGCS1, HMGCS2, LSS, MSMO1, NSDHL, SC5D, SQLE |
| **Cholesterol Biosynthesis I** | 2.38 | 0.615 | 2.828 | CYP51A1, DHCR24, FDFT1, LSS, MSMO1, NSDHL, SC5D, SQLE |
