# Supplementary material for: Lipidomic and transcriptomic remodeling underlies early retinal pathogenesis in dry age-related macular degeneration
Source: Cell Death Dis. 2026 Aug 7;17(1):937. doi: 10.1038/s41419-026-09147-5 (PMC13736989; doi:10.1038/s41419-026-09147-5)
Supplement: Supplementary file 3 [file 41419_2026_9147_MOESM15_ESM.docx]

| **Compound** | **Quantifier parent>daughter MRM** |
| --- | --- |
| AAd8 | 311.34>267.34 |
| FA32:6 | 467.513>389.46 |
| FA34:6 | 495.513>417.37 |
| FA38:6 | 551.50>473.37 |
| 32:6monoHDHA | 483.50>385.50 |
| 34:6monoHDHA | 511.50>413.50 |
| 36:6monoHDHA | 539.50>441.50 |
| 38:6monoHDHA | 567.50>469.50 |
| 4b-OH | 385.277>367.431 |
| 20-OH | 385.277>95.127 |
| 7b-OH | 385.277>367.431 |
| Cholestenone | 385.277>109.164 |
| 5b-6b-epoxy-cholestanol | 385.277>367.431 |
| Cholesterol-d7 | 376.277>160.981 |
| Cholesterol | 369.277>161.031 |
| 7-keto Cholesterol | 401.213>95.127 |

**Supplementary Table 4.** Multiple Reaction Monitoring (MRM) transitions used for the quantification of VLC‑PUFAs. MRM transitions are annotated as precursor ion → fragment ion (m/z) in the right‑hand column and correspond to the compounds listed in the left‑hand column. Deuterated internal standards were employed for normalization: cholesterol‑d7 for cholesterol species and arachidonic acid‑d8 (AA‑d8) for VLC‑PUFA compounds.
