# Supplementary material for: Lipidomic and transcriptomic remodeling underlies early retinal pathogenesis in dry age-related macular degeneration
Source: Cell Death Dis. 2026 Aug 7;17(1):937. doi: 10.1038/s41419-026-09147-5 (PMC13736989; doi:10.1038/s41419-026-09147-5)
Supplement: Supplementary file 4 [file 41419_2026_9147_MOESM16_ESM.docx]

| **Compound** | **Precursor ion (*m/z*)** | **Neutral Loss (*m/z*)** |
| --- | --- | --- |
| **PC** | **184.10** | **-** |
| **PE** | **-** | **141.10** |
| **PS** | **-** | **185.10** |
| **SM** | **184.10** | **-** |
| **CER** | **264.30** | **-** |

**Supplementary Table 5:** MS/MS Scans for Semi-Quantification of Phospholipids. Neutral loss and parent ion scanning strategies were used for the separation and semi-quantification of phospholipid and sphingolipid classes. Each precursor ion or neutral loss scan (m/z) noted, are the fragmented ions of the specific lipid headgroups that are generated during collision induced dissociation (CID); this allowed for separation, classification, and relative quantification of individual phospholipid species. For each phospholipid, the resulting daughter ions were verified using LIPID MAPS public database (https://www.lipidmaps.org/) and by in-house comparison using synthetic standards. Phosphatidylcholine (PC) and sphingomyelin (SM), which share a common diagnostic ion at m/z 184.1, were resolved based on chromatographic retention times, with PC eluting at ~8 min and SM at ~10 min, as verified using synthetic standards during method development. PC was analyzed by MS scanning (5–20 min, ES+, cone voltage 30 V) followed by parent ion scanning of m/z 184.10 corresponding to the phosphocholine head group. Phosphatidylethanolamine (PE) and phosphatidylserine (PS) species were analyzed using neutral loss scans of m/z 141.1 and m/z 185.1, respectively, reflecting loss of their characteristic head groups. Ceramides (CER) were analyzed using MS/MS and parent ion scanning of m/z 264.3, corresponding to loss of the amide‑bound fatty acyl chain.
