# Supplementary material for: Lipidomic and transcriptomic remodeling underlies early retinal pathogenesis in dry age-related macular degeneration
Source: Cell Death Dis. 2026 Aug 7;17(1):937. doi: 10.1038/s41419-026-09147-5 (PMC13736989; doi:10.1038/s41419-026-09147-5)
Supplement: Supplementary file 5 [file 41419_2026_9147_MOESM1_ESM.docx]

Roy et al., CDDIS-26-200-T

**SUPPLEMENTAL INFORMATION**

**Five Supplementary Tables**

**10 Supplementary Figures**

**Supplementary Table Legends**

**Table S1.** List of genes used in the survey of signaling pathways analyzed in Figure 1.

**Table S2.** Representative significantly enriched IPA canonical pathways identified from DEG (RPE of CLIC4-KO vs. Ctrl) analysis. This analysis is part of the study reported in (1) but was not presented in the previous publication. For each pathway, the -log(Benjamini–Hochberg–adjusted p-value), ratio of DEGs to total genes in the pathway, activation z-score, and contributing genes are provided. Genes discussed in the text are labeled in red.

**Table S3.** Complete list of canonical pathways enriched among DEGs identified in the rods of KO compared with Ctrl samples, as determined by IPA. For each pathway, the -log(p-value), activation z-score, and ratio of DEGs to total genes in the pathway are provided.

**Table S4.** Multiple Reaction Monitoring (MRM) transitions used for the quantification of VLC‑PUFAs. MRM transitions are annotated as precursor ion → fragment ion (m/z) in the right‑hand column and correspond to the compounds listed in the left‑hand column. Deuterated internal standards were employed for normalization: cholesterol‑d7 for cholesterol species and arachidonic acid‑d8 (AA‑d8) for VLC‑PUFA compounds.

**Table S5:** MS/MS Scans for Semi-Quantification of Phospholipids. Neutral loss and parent ion scanning strategies were used for the separation and semi-quantification of phospholipid and sphingolipid classes. Each precursor ion or neutral loss scan (m/z) noted, are the fragmented ions of the specific lipid headgroups that are generated during collision induced dissociation (CID); this allowed for separation, classification, and relative quantification of individual phospholipid species. For each phospholipid, the resulting daughter ions were verified using LIPID MAPS public database (https://www.lipidmaps.org/) and by in-house comparison using synthetic standards. Phosphatidylcholine (PC) and sphingomyelin (SM), which share a common diagnostic ion at m/z 184.1, were resolved based on chromatographic retention times, with PC eluting at ~8 min and SM at ~10 min, as verified using synthetic standards during method development. PC was analyzed by MS scanning (5–20 min, ES+, cone voltage 30 V) followed by parent ion scanning of m/z 184.10 corresponding to the phosphocholine head group. Phosphatidylethanolamine (PE) and phosphatidylserine (PS) species were analyzed using neutral loss scans of m/z 141.1 and m/z 185.1, respectively, reflecting loss of their characteristic head groups. Ceramides (CER) were analyzed using MS/MS and parent ion scanning of m/z 264.3, corresponding to loss of the amide‑bound fatty acyl chain.

**Supplementary Figure Legends**

**Fig. S1. Transcriptomic Changes in Lipid Metabolism, Lipid Transport, Master Regulators of Homeostasis, and Mitochondrial Networks in CLIC4-KO RPE**
Heatmaps depicting fold changes in mRNA levels of genes involved in lipid metabolism, lipid transport, and master regulators of cellular homeostasis (A), as well as genes related to mitochondrial and peroxisomal function (B), based on bulk RNA-seq of CLIC-KO versus control (Ctrl) RPE cells. N = 3 biological replicates per group. Statistically significant changes (FDR ≤ 0.05) are indicated with asterisks in (A). (C) Representative enriched IPA canonical pathways from RNA-seq comparing CLIC4-KO and Ctrl RPE cells. P-values were adjusted for multiple testing using the Benjamini–Hochberg method.

**Fig. S2. Altered PC Species in Retina and RCS in RPE^∆^*^Clic4^* mice**
Bar graphs showing changes in all PC species measured in retina (A) and RCS (B), presented for mixed-sex cohorts (♀♂, N = 6 each) and stratified by female (♀) and male (♂) groups (N = 3 each). Data represent mean ± SD. Statistical significance was assessed by two-tailed Student’s t-test (*p ≤ 0.05, **p < 0.01). Source data of (A, B) are provided as a Source Data file.

**Fig. S3. Altered PE Species in Retina and RCS of RPE^∆^*^Clic4^* mice**
Bar graphs showing changes in all PE species measured in retina (A) and RCS (B), presented for mixed-sex cohorts (♀♂, N = 6 each) and stratified by female (♀) and male (♂) groups (N = 3 each). Data represent mean ± SD. Statistical significance was assessed by two-tailed Student’s t-test (*p ≤ 0.05, ** p < 0.01). Source data of (A, B) are provided as a Source Data file.

**Fig. S4. Altered PS Species in Retina and RCS of RPE*^∆Clic4^* mice**
Bar graphs showing changes in all PS species measured in retina (A) and RCS (B), presented for mixed-sex cohorts (♀♂, N = 6 each) and stratified by female (♀) and male (♂) groups (N = 3 each). Data represent mean ± SD. Statistical significance was assessed by two-tailed Student’s t-test (*p ≤ 0.05, **p < 0.01). Source data of (A, B) are provided as a Source Data file.

**Fig. S5. Elovanoid Biosynthesis and Changes in PUFA, Cholesterol, and Oxysterol Profiles in Retina and RCS of RPE*^∆Clic4^* mice**
(A) Schematic of the elovanoid biosynthesis pathway.
(B, C) Changes in PUFAs and hydroxy-PUFAs in female, male, and mixed-sex retina and RCS cohorts (N = 3 per sex).
(D, E) Bar graphs showing changes in indicated VLC-PUFA species in retina (E) and RCS (F), presented for mixed-sex cohorts (N = 6 each) and stratified by female and male groups (N = 3 each). Data represent mean ± SD. Statistical significance was assessed by two-tailed Student’s t-test (*p < 0.01). Source data of (B-E) are provided as a Source Data file.

**Fig. S6. Altered Sphingomyelin Species in Retina and RCS of RPE*^∆Clic4^* mice**
(A) Heatmaps display fold changes in **sphingomyelin** species in retina and RCS tissues from KO mice compared to Ctrl. The x-axis represents the number of double bonds, and the y-axis shows the carbon chain length of each lipid species. Fold-change values are indicated within each heatmap cell. **(B, C)** Bar graphs showing all sphingomyelin species measured in retina (B) and RCS (C), presented for mixed-sex cohorts (N = 6 each) and stratified by female and male groups (N = 3 each). Data represent mean ± SD. Statistical significance was assessed by two-tailed Student’s t-test (*p ≤ 0.05, **p < 0.01). Source data of (B, C) are provided as a Source Data file.

**Fig. S7. Altered Ceramide Species in Retina and RCS of RPE^∆^*^Clic4^* mice**
Bar graphs showing all ceramide species measured in retina (A) and RCS (B), presented for mixed-sex cohorts (N = 6 each) and stratified by female and male groups (N = 3 each). Data represent mean ± SD. Statistical significance was assessed by a two-tailed Student’s t-test (*p ≤ 0.05, **p < 0.01). Source data of (A, B) are provided as a Source Data file.

**Fig. S8. Altered Cholesterol and Oxysterol Species in Retina and RCS of RPE^∆^*^Clic4^* mice**
Bar graphs showing all cholesterol and oxysterol species measured in retina (A) and RCS (B), presented for mixed-sex cohorts (N = 6 each) and stratified by female and male groups (N = 3 each). Data represent mean ± SD. Statistical significance was assessed by a two-tailed Student’s t-test (*p ≤ 0.05, **p < 0.01). Source data of (A, B) are provided as a Source Data file.

**Fig. S9. Integration and Clustering of Retinal scRNA-Seq Data of RPE^∆^*^Clic4^* mice**
t-SNE projection of merged retinal single-cell RNA-seq data colored by cluster identity (left) and genotype (right). The integration effectively removed genotype-driven batch effects, with minimal differences between samples. Clustering matches the UMAP plot shown in Fig. 5A.

**Fig. S10 Visualization and Quantification of Retinal 4-HNE Immunoreactivity.**

(A, C) Representative retinal sections immunostained for 4-HNE in 3-month-old (A) and 6-month-old (B) Ctrl and KO mice. (B, D) Layer-stratified quantification of 4-HNE immunoreactivity from (A) and (C), respectively. Staining intensity (arbitrary fluorescence units, AFU) was measured in the IS/OS and outer nuclear layer (ONL). Data are presented as mean ± SD; n = 3 female mice per genotype, with 9 and 12 image fields quantified for Ctrl and KO groups, respectively. Statistical significance was determined by a two-tailed Student's t-test. Source data for (B) and (D) are provided as a Source Data file. *p ≤ 0.05.

Reference
